# Severe Radiation-Induced Brachial Plexopathy: A Case Report on Radiation Toxicity in a Patient With Invasive Ductal Carcinoma

**DOI:** 10.7759/cureus.73043

**Published:** 2024-11-05

**Authors:** Kathleen Waeldner, Christine Chin, Philip Gilbo

**Affiliations:** 1 Department of Radiation Oncology, Larner College of Medicine at the University of Vermont, Burlington, USA; 2 Department of Radiation Oncology, Nuvance Health, Norwalk, USA

**Keywords:** atm mutation, brachial plexopathy, invasive ductal breast carcinoma, palb2 mutation, radiation-induced brachial plexopathy

## Abstract

The ataxia-telangiectasia mutated (*ATM*) gene is an important regulator of cell checkpoint signaling and the repair of double-stranded breaks. When the *ATM* gene is mutated or damaged, cells are less capable of responding to damage induced by radiation therapy (RT). Here, we present a case of a 50-year-old woman with stage IIIA invasive ductal carcinoma of the left breast who had genetic testing revealing pathogenic *ATM* mutations (c.5290del and c.4396C>G) and a *PALB2* mutation (c.1619dup). While guidelines suggest that adjuvant radiation therapy is safe for patients with *ATM* mutations, this patient experienced severe radiation-induced toxicities, including brachial plexopathy. These *ATM* mutations have not previously been described as imparting severe radiation-associated toxicities.

## Introduction

The ataxia-telangiectasia mutated (*ATM*) gene, an oncosuppressor involved in DNA repair response pathways, is an important regulator of cell checkpoint signaling and the repair of double-stranded breaks by phosphorylating downstream proteins such as p53 and BRCA1, which are implicated in breast cancer. When the *ATM* gene is mutated or damaged, cells are less capable of responding to double-stranded DNA breakage or DNA damage induced by radiation therapy (RT) [1]. Multiple studies have shown that patients with *ATM* mutations, specifically the 5557 G>A single-nucleotide polymorphism (SNP), are associated with increased RT sensitivity [2-5].

Despite this radiosensitivity, adjuvant radiation remains safe for most breast cancer patients who harbor *ATM* mutations [6]. The American Society for Radiation Oncology (ASRO) recommends radiation therapy for patients with breast cancer when clinically indicated; variants of uncertain significance (VUS) should be considered normal regarding radiation decision-making [7].

## Case presentation

A 50-year-old woman presented with the American Joint Committee on Cancer (AJCC) eighth edition anatomic stage IIIA invasive ductal carcinoma of the left breast, cT2N1M0, grade 2, estrogen receptor (ER)-positive, progesterone receptor (PR)-positive, and human epidermal growth factor receptor 2 (HER2)-negative. She had no significant predisposing history for risk for breast cancer. On genetic testing with Invitae 47 genetics panel, she was found to harbor pathogenic heterozygosity for *ATM* variant c.5290del (p.Leu1764TyrFs*12), pathogenic heterozygosity for *PALB2* variant c.1619dup (p.Asn540Lysfs*38), and a second heterozygous *ATM* variant, c.4396C>G (p.Arg1466Gly), which was of uncertain significance.

She received treatment with neoadjuvant dose-dense adjuvant Adriamycin and Cytoxan followed by trastuzumab (ddAC-T). Approximately one month after completing chemotherapy, she underwent a left breast lumpectomy and sentinel lymph node biopsy demonstrating stage IA residual disease, ypmT1cN0Mx. Following multidisciplinary discussion and approximately eight weeks after completing chemotherapy, she received adjuvant radiotherapy consisting of 5000 centigrays (cGy)/25 fractions (fx) to the left breast and regional nodes (axilla, supraclavicular, and internal mammary) plus 1000 cGy/4fx to the tumor bed as a boost. Two weeks after completing radiation, she started letrozole. About four months after therapy, she began to experience a fullness sensation in the supraclavicular area and significant breast shrinkage. She began lymphedema therapy. Despite therapy, her symptoms continued to progress. By 10 months, she had developed severe back pain and "burning" pain in her left axilla with occasional bilateral shooting pains in the upper extremities. Body imaging (spine MRI, PET, and CT) at that time showed no evidence of recurrence, though RT-related fractures were observed in the ribs. She was placed on a prednisone taper and pain medicine and responded well to this intervention. At 13 months, she developed symptomatic premature atrial contractions. She started on cardiac medications and improved. During this timeframe, she continued with lymphedema therapy, and her symptoms waxed/waned. She continued to experience sensations consistent with bilateral cervical radiculopathy, though around this time a tingling sensation developed in her left upper extremity. Breast examination continued to show marked shrinkage on the left with the development of telangiectasias around this time.

**Figure 1:**
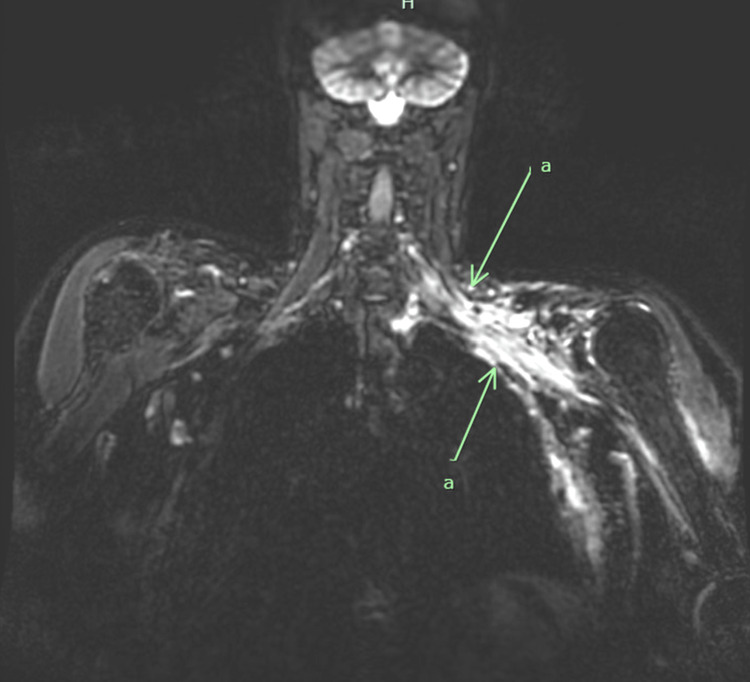
Coronal Chest MRI Without Contrast of Brachial Plexopathy (a) T2 hyperintense signal tracking along the entire course of the left brachial plexus extending from the trunk along the neurovascular bundle to the left axilla

By 20 months, lymphedema symptoms had significantly improved, but she began to experience neurologic symptoms in the distal left upper extremity, and this led to her formal diagnosis of radiation-induced brachial plexopathy. Dedicated MRI demonstrated edema and diffuse, confluent, T2 hyperintense signal tracking along the entire course of the left brachial plexus extending from the trunk along the neurovascular bundle to the left axilla (Figure 1). She was started on hyperbaric oxygen (30 treatments), as well as pentoxifylline with vitamin E with no measurable impact. Overall, the symptoms of the affected limb continued to wax and wane, though inexorably worsened, and she received supportive care for her symptoms. The utilization of tetrahydrocannabinol (THC) edible gummies provided relief of neuropathy on top of typical neuropathic medications, and she was eventually started on neuromuscular and muscular electrical stimulation, which significantly helped with edema in the limb, improved strength in the proximal limb, and improved her range of motion. The breast and chest continued to progress with severe fibrosis and telangiectasias. By two years, her examination had stabilized. At approximately 3.5 years out from the end of radiation therapy, she has no evidence of disease recurrence. She continues to consider prophylactic procedures to address her risks in other organs from the genetic mutations.

The patient was treated at Nuvance Health by Dr. Philip Gilbo; Kathleen Waeldner is a medical student who rotates through Nuvance through an institutional agreement with the University of Vermont Larner College of Medicine. Dr. Christine Chin participates in Nuvance Department of Radiation Oncology reviews and participated in the development of this paper.

## Discussion

Homozygous mutations in the *ATM* gene cause ataxia-telangiectasia, an autosomal recessive neurodegenerative disease characterized by gradual cerebellar cortical atrophy, telangiectasia of the eyes and facial skin, immune deficiency, premature aging, increased likelihood of developing multiple types of malignancies, and heightened sensitivity to radiation therapies [7]. The *ATM* gene, an oncosuppressor involved in DNA repair response pathways, functions in the double-stranded DNA repair pathway and regulates downstream proteins such as p53 and BRCA1. When the *ATM* gene, located on chromosome 11q22-23, is mutated or damaged, these downstream genes become less effective. Without *ATM* functionality, cells are less capable of responding to double-stranded DNA breakage [1,8,9]. This loss provides the primary mechanism for both increased malignant potential and radiation sensitivity as the double-stranded break is the primary mechanism for cell death following conventional radiation therapy. Many mutations within this gene are possible, though the phenotypic results are heterogeneous. Some clear examples of increased radiation sensitivity involve the 5557 G>A single-nucleotide polymorphism (SNP) mutation, yet others have not been associated with any ill effects [2-5].

In 2020, the American Society for Radiation Oncology (ASRO) published a guide with recommendations regarding genetic testing prior to radiation therapy. Variants of uncertain significance (VUS) are to be considered nondeleterious until functional genomic data emerge to demonstrate otherwise, and that possession of germline alterations in a single copy of a gene critical for radiation damage responses does not necessarily equate to increased risk of radiation-induced toxicity [7]. Further, McDuff et al. reported that adjuvant radiation is safe for most breast cancer patients who harbor *ATM* variants [6].

In this patient, the decision was made to proceed with a typical radiation treatment given there are no noted deleterious outcomes within the known literature. She unfortunately developed severe radiation effects including rib fractures, fibrosis, breast shrinkage, marked telangiectasias, possible cardiac arrhythmias, edema, and, notably, brachial plexopathy leading to limb paralysis. There are aspects of this patient's case that are notable. She had both a "pathogenic" *ATM* mutation, a VUS *ATM* mutation, and a *PALB2* mutation. We hypothesize that the increased radiation sensitivity could be due to the fact that two mutations were present instead of one, the combination of the *ATM* and *PALB2* mutations, or stemming from the individual mutations in either of the *ATM* genes.

For example, Iannuzzi et al. found a significant correlation between *ATM* mutation status and the development of grade 3-4 subcutaneous late effects. Within this study, the patients who developed high-grade late sequelae had two *ATM* mutations. Comparatively, patients with a single *ATM* mutation did not develop severe subcutaneous reactions [3].

Further, *PALB2* is another oncosuppressor that acts as a mediator between BRCA2 and BRCA1, an essential step in homologous recombination repair [10]. Several studies have demonstrated that *ATM* and *PALB2* mutations confer a moderate risk of developing breast cancer [11-15]. However, unlike *ATM* mutations, there are no known radiation sensitivities associated with *PALB2*. We were unable to identify any literature regarding increased sensitivity to radiation therapy in patients who harbor both an *ATM* mutation and a *PALB2* mutation, but given that both these genes assist in DNA repair, it is reasonable to consider that the effects of their mutations may compound each other to increase radiation sensitivity as seen in this case [1,16].

Finally, it is reasonable to consider that, given the relative infrequency of these reported mutations, one of them is responsible for the apparent increased sensitivity given the incomplete understanding of the radiation sensitivity imparted by different *ATM* mutations.

Ultimately, given the lack of strong evidence for increased radiation sensitivity outside of particular mutations (Table 1), the consensus remains that individuals with pathogenic or VUS mutations continue to receive the benefits of radiation therapy in the paradigm of their breast cancer treatment.

**Table 1:** Current Literature Findings of Toxicities Associated With ATM Mutations VUS, variants of uncertain significance; *ATM*, ataxia-telangiectasia mutated

Study	*ATM*-specific mutation	Toxicity findings
Nepomuceno et al. [16]	Multiple	No difference in toxicity, pathogenic versus VUS
Modlin et al. [17]	5557G>A	Grade 3 fibrosis
Andreassen et al. [18]	5557G>A	Grade 3 fibrosis and telangiectasias
Iannuzzi et al. [3]	4138C>G, 2632A>C, 7392C>T, 6088A>G, and 735C>T	Grade 3 confluent moist desquamation, grade 3 fibrosis, grade 4 soft tissue necrosis, and grade 3 telangiectasias

## Conclusions

While significant literature exists regarding the link between *ATM* mutations, breast cancer incidence, and radiation sensitivity, this paper reports on a severe case of radiation-induced brachial plexopathy and other likely radiation-related toxicities in the setting of *ATM* variant c.5290del (p.Leu1764TyrFs*12) and *ATM* variant c.4396C>G (p.Arg1466Gly) in the setting of *PALB2* variant c.1619dup (p.Asn540Lysfs*38). These *ATM* mutations have not previously been described as imparting severe radiation-associated toxicities. Radiation oncologists should exercise caution in this clinical scenario.
